# ‘Honeycrisp’ Bitter Pit Response to Rootstock and Region under Eastern New York Climatic Conditions

**DOI:** 10.3390/plants10050983

**Published:** 2021-05-14

**Authors:** Daniel J. Donahue, Gemma Reig Córdoba, Sarah E. Elone, Anna E. Wallis, Michael R. Basedow

**Affiliations:** 1 Eastern New York Commercial Horticulture Program, Cornell Cooperative Extension, Cornell University, 3357 Route 9W, Highland, NY 12528, USA; ser37@cornell.edu; 2 IRTA Fruitcentre, Tree Fruit Production, PCiTAL, Park of Gardeny, Fruitcentre Building, 25003 Lleida, Spain; gemma.reig@irta.cat; 3 Cooperative Extension, Michigan State University, 775 Ball Ave NE, Grand Rapids, MI 49503, USA; wallisa2@msu.edu; 4 Eastern New York Commercial Horticulture Program, Cornell Cooperative Extension, Cornell University, 6064 Route 22 Suite 5, Plattsburgh, NY 12901, USA; mrb254@cornell.edu

**Keywords:** PLSR model, B.9, M.26, M.9, environment, fruit quality

## Abstract

There are still unknown factors at play in the causation of bitter pit in ‘Honeycrisp’ as well as in other apple varieties. To investigate some of these factors, we conducted a survey of 34 ‘Honeycrisp’ orchard blocks distributed across two disparate production regions in eastern New York State, representing a variety of rootstocks, over three growing seasons. Weather, soil, horticultural traits, fruit quality traits, pick timing, leaf and peel minerals were evaluated for their impact on bitter pit (BP) incidence; factors were further evaluated for their interaction with region and rootstock. ‘Honeycrisp’ trees on B.9 rootstock were smaller but with comparable terminal shoot growth when compared to those on M.26 and M.9 rootstocks. B.9 fruits, which had similar fruit size to M.26 and M.9 and had good fruit quality at harvest and after storage, were much less likely to express bitter pit symptoms compared to M.9 and M.26 rootstocks. Not all traits evaluated individually correlated significatively with bitter pit incidence after a period in storage. Depending on rootstock and region, the correlation could be significant in one situation, with no correlation at all in another. In this study, peel Mg/Ca ratio and peel Ca correlated with BP for all three rootstocks, with the strongest correlations associated with the M.9 clones. These same traits correlated with BP for both regions. Pick timing had a significant influence on BP incidence following storage, with later picks offering better bitter pit storage performance. While excessively large fruits, those in the 48 and 56 count size categories, were found to be highly susceptible to BP regardless of rootstock, B.9 BP fruit susceptibility for smaller sizes was found to be size neutral. A PLSR prediction model for each rootstock and each region showed that different variables correlated to BP depending on the situation. Thus, the results could suggest that in addition to the variables considered in this study, there are other less studied factors that can influence the expression of BP symptoms. We strongly suggest that rootstock BP performance be considered a critical parameter when planning a commercial ‘Honeycrisp’ orchard and be evaluated in rootstock breeding and development programs prior to wide commercial release.

## 1. Introduction

*Malus* × *pumila* Borkh ‘Honeycrisp’ is a variety of major economic significance and has achieved widespread commercial success in North America and Europe since its introduction by the University of Minnesota apple breeding program in 1991 [1]. Currently, ‘Honeycrisp’ is the third most produced apple variety in the USA, after ‘Gala’ and ‘Red Delicious’. All three varieties comprise 48% of all apple production in 2020 [2]. ‘Honeycrisp’ is a high-value bi-color apple that became popular for its unique fruit quality characteristics and palatability at harvest (juicy, crispy, and strong flavored) that are maintained during storage [3]. However, it has developed a reputation for being a challenging apple to grow. In fact, it presents growers and marketers with several production and storage difficulties, such as alternate bearing, sunburn, bitter pit disorder (BP), soft scald, soggy breakdown, leather blotch and wrinkly skin [3,4,5,6,7]. The correct pedigree of ‘Honeycrisp’, ‘Keepsake’ × MN1627 (‘Duchess of Oldenburg’ × ‘Golden Delicious’) [8] may help to explain its susceptibility to bitter pit. ‘Golden Delicious’ is a bitter pit-susceptible apple, as reported in previous studies [9,10].

Bitter pit (BP) is a calcium-related physiological disorder that expresses as dark sunken necrotic lesions in the first few millimeters of the skin and flesh, typically near the calyx end of the fruit, and is the result of a complex interaction between fruit physiological factors, as well as certain environmental and horticultural conditions. Historically, more than 50% of fruit from young ‘Honeycrisp’ plantings is unmarketable due to bitter pit [5,11] and pack-out percentages can be even less after long-term storage. The commercial conditioning treatment of 7 days at 10 °C used to reduce risk of soft scald and soggy breakdown development, as well as recommended storage temperature of 3 °C exacerbate losses due to bitter pit [12]. Despite this, an increasing number of hectares continue to be planted each year, and many blocks have yet to reach full bearing, increasing market supply. Therefore, as ‘Honeycrisp’ production continues to expand, particularly in North America, knowing as many variables as possible related to bitter pit will be necessary for the economic sustainability of this variety. 

Producers have traditionally attempted to mitigate the expression of BP symptoms through the foliar application of calcium, with variable success. Many studies carried out in ‘Honeycrisp’ and other apple varieties such as ‘Golden Delicious’, ‘Fuji’ and ‘Braeburn’ contributed to the development of strategies to reduce bitter pit incidence [10,13] and models or methods to predict bitter pit before harvest [12,14,15,16]. Most are related to the mineral nutrition and bitter pit incidence relationship. Two studies reported a clear, positive relationship between potassium-to-calcium ratio and bitter pit incidence [17,18] (Valverdi et al., 2021; Fazio et al., 2020). However, mineral content is not the only criterion for the occurrence of bitter pit and does not always explain the presence or absence of symptoms in a particular orchard [13]. Other factors such as early harvest, poorly drained fine-textured soils, light cropping, excessive tree vigor, excessive nitrogen nutrition, moisture stress, low boron, vigorous shoot growth, weather and soil conditions (hot and drying conditions, low soil pH), year, local environment, hormones, rootstock selection, foliar calcium programs and tree age can contribute to increase bitter pit risk [14,19,20] and to explain the enormous variation in bitter pit incidence observed among commercial plantings. A study that compared B.9, M.9, G.41, and G.890 rootstocks reported a strong effect of rootstock on nutrient composition and partitioning as well as overall fruit quality and disorder incidence in ‘Honeycrisp’ apple [18].

The aim of this study was to broadly examine potential contributors to the large variation observed in the rate of bitter pit incidence on ‘Honeycrisp’ in the New York State climatic environment. We focused on rootstock and region, analyzing weather, soil, horticultural and fruit quality variables, using multivariate and binomial distribution analysis techniques.

## 2. Materials and Methods

### 2.1. Plant Material and Site Description

**Figure 1:**
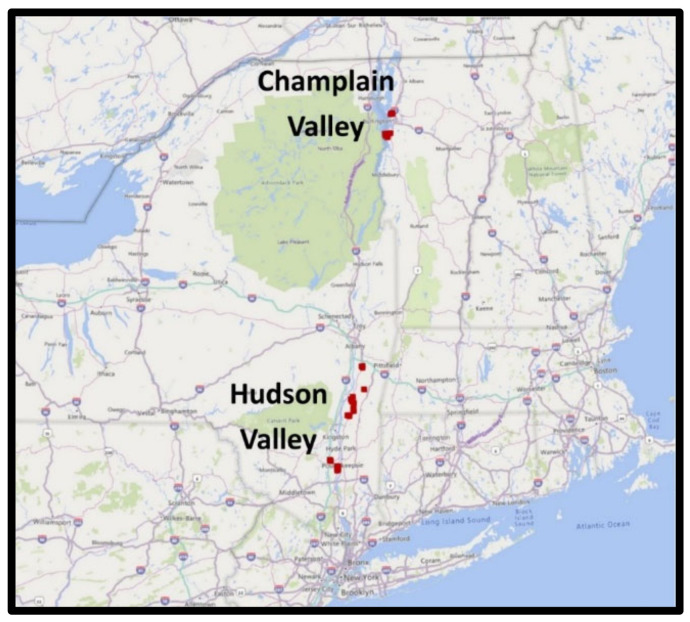
Map of the study domain in Eastern New York State, USA.

From two distinct apple production regions within Eastern New York (Hudson Valley (HV), and the Champlain Valley (CV), thirty-four commercial ‘Honeycrisp’ orchards in Eastern New York State (NYS), USA, were initially selected randomly in the spring of 2016 on the basis of having recognized commercial success, a modern vertical axis or tall spindle planting system, and a modest tree size that would facilitate data collection. Larger tree forms such as semi-dwarf central leader on wider spacings were excluded on the basis of declining commercial viability (Table 1, Figure 1). Twenty orchards were located within the Hudson Valley (HV) in the southeast and fourteen were located within the Champlain Valley (CV), approximately 300 km to the north. Orchard age ranged from 4th leaf to 25th leaf, tree training was uniformly central leader, but planting system design included a variety of planting densities from tall spindle at 1 m × 3.6 m to vertical axis at 2.4 m × 4.9 m. The pest management program for each orchard was managed by the local grower according to accepted commercial practices with the exclusion of foliar calcium and Prohexadione calcium sprays. Pruning practices and crop load management were managed by the grower according to commercial standards. The experimental unit within each orchard was defined as six contiguous trees of uniform presentation deemed to be representative of the orchard as a whole. Beyond this initial selection, data collection from the experimental unit was restricted to the trees within the unit and not considered to be representative of the orchard as a whole. Approximately half of the selected orchards were irrigated by trickle systems from local ponds and wells. The remaining half had access to solid-set overhead irrigations systems grower, deployed on an as-needed basis during extended periods of dry weather. The irrigation strategies present in this survey study are representative of commercial grower practices in both growing regions.

### 2.2. Weather Conditions

For the years 2016–2018, meteorological data (degree days and rainfall) was downloaded from the nearest meteorological station for each orchard, often on-farm in close proximity. Degree day (DD) was calculated as the difference between the daily mean temperature and a base temperature of 3.9 °C. Then, annual rainfall (mm) and annual DD accumulation (from 1 January to 30 June and from 1 May to 30 June) were calculated (Table 2). The two regions of this study are separated by approximately 320 km and have different climates. For the Hudson Valley, the average winter temperature is −2.1 °C and average summer temperature is 19.3 °C. Average annual precipitation is 73.1 cm. For the Champlain Valley, the average winter temperature is −5.6 °C and average summer temperature is 17.9 °C. Average annual precipitation is 82.4 cm.

### 2.3. Soil Conditions

A Watermark SS200 solid-state electronic soil water tension measurement sensor (Irrometer Company, Riverside, CA, USA) with a measurement range of 0–200 kilopascals was installed per manufacturer recommendations at a soil depth of 20 cm in the center of each 6-tree experimental unit. Measurement data was taken weekly for the period full bloom to harvest using a hand-held Watermark meter. A soil sample of 12 cores (both sides of each tree) from the depth range of 2.5–20 cm was taken in each experimental unit in 2016 and 2017. In 2018, a single sample of 20 cm × 20 cm × 20 cm was taken from a single site within each experimental unit as specified by the Cornell Soil Health Testing protocol.

Rapid Soil Texture: The non-organic, solid material in soils is composed of a mixture of mineral particle sizes, the relative amounts of which determine the soil texture. Textural class affects many of the important physical, biological, and chemical processes in the soil, but is not easily altered by management practices because it changes little over time. Although soil texture itself is not a soil health indicator per se, knowledge of the textural class informs the interpretation of soil health indicators. This Rapid Texture procedure involves dispersion of soil particles using sodium hexametaphosphate followed by the (1) isolation of the sand fraction using a 0.053 mm sieve, and (2) separation of the silt and clay fractions by settling. 

Available Water Capacity: Available water capacity is the amount of plant-available water the soil can store. In the field, a soil is at the upper end of soil water storage when water that it cannot hold against the force of gravity has—this is called ‘field capacity.’ The lower end of the range is referred to as the ‘permanent wilting point,’ which occurs when only hygroscopic water remains (i.e., water tightly held so tightly onto soil particle surfaces that it is inaccessible by plant roots). Available water capacity is determined from measuring the soil’s water content at both field capacity and permanent wilting point in the lab and calculating the difference. 

Wet Aggregate Stability: Wet aggregate stability is a measure of the extent to which soil aggregates resist falling apart (i.e., slaking) when wetted and exposed to the force of rain drops. This physical soil property is measured using the Cornell Sprinkle Infiltrometer, which rains for 5 min (delivering 1.25 cm of water) on a sieve containing a known weight of soil aggregates between 0.25 mm and 2.0 mm. Unstable aggregates slake and pass through the sieve, while the fraction of soil remaining on the sieve is used to calculate the percent wet aggregate stability. Soils with high wet aggregate stability are more resistant to water and wind erosion and show better overall soil health (e.g., infiltration, root growth, biological activity). 

### 2.4. Horticultural Assessments 

Within each experimental unit and for each year the following horticultural measurements were recorded, and samples taken: trunk diameters were measured at a point 30 cm from the ground and used to calculate trunk cross-sectional area (TCSA) in cm^2^ in the spring. Ten random terminal shoots were selected per tree, five per side, following terminal bud set in the late summer, and measured for length (mm). A minimum of 20 terminal shoot leaves of moderate age were sampled from trees in each experiment unit for nutrient analysis at approximately 80 days post-full bloom. Apples were sampled for peel mineral analysis at approximately five weeks before first harvest. A spiral peeler (Apple Mate 861, Norpro, Moodus, CT, USA)) was used to produce peel of 0.8 mm thickness with the calyx half separated from the stem half. Two fruits per tree were sampled from each experimental unit for a total of twelve. Peels from each experimental unit were pooled with calyx and stem-end tissue analyzed separately for mineral content. Results presented are from the calyx-end samples.

### 2.5. Plant Tissue and Soil Mineral Analysis

Peel and leaf tissue mineral analysis: Samples were digested with nitric and perchloric acids using the Vulcan 84 automated digestion system (Questron Technologies Cor. Mississauga, ON, Canada). Approximately 0.3 to 1.0 grams of sample were weighed into 50 mL Teflon containers plus 0.25 mL of 80 µg per mL of yttrium. This was used as an internal standard. The digestion system automatically added (using syringe pumps) 5.0 mL of 67–70% nitric acid (OmniTrace, Delray Beach, FL, USA) plus 3.0 mL of environmental grade 70% perchloric acid (GFS chemicals, Columbus, OH, USA). In this system, the samples were heated to 110 °C over 40 min and held for 60 min. The temperature increased to 160 °C over 20 min and was held for 15 min. An additional 1.0 mL of nitric acid was added and the samples were heated an additional 20 min at 160 °C. After cooling, 20.0 mL of 18 mg water was added. The solutions were then analyzed using an axial viewed ICP-OES. (Spectro Arcos FHE12, Kleve, Germany). Results are not corrected for moisture content. 

Organic matter content: The pool of organic matter (OM) in soil includes the carbon-containing solid materials which are, or are derived from, living organisms including plants and soil microorganisms. Soils with high OM content tend to require lower inputs of nutrients and are more resilient to drought and extreme rainfall. The percent OM was determined by measuring the mass loss on ignition (LOI) at 500 °C in a furnace. At these extreme temperatures, carbonaceous materials are burned off (i.e., oxidized to carbon dioxide (CO_2_)), while non-organic mineral materials remain. 

pH and soil mineral testing: A traditional soil fertility test analysis designed for application in the Northeastern USA was used to determine soil pH and estimate plant nutrient availability. Nutrient analysis was performed using a Modified Morgan (MM) extraction and reported in available nutrients. Samples were sieved past 2 mm and placed in labeled paper cups, dried at 50 °C before extraction. For the MM extraction, soil was extracted at a 1:4 soil/solution ratio with Modified Morgan solution [0.62 N NH_4_OH + 1.25 N CH_3_COOH] buffered at pH 4.80 with the filtrate subsequently analyzed by ICP for Ca, Mg K, Fe Al, Mn, and Zn. Soil pH is a measure of hydronium ion (H_3_O+, or more commonly the H+) activity in the soil solution. Soil pH influences many facets of crop production and soil chemistry, including availabilities of nutrients and toxic substances, activities and nature of microbial populations, and activities of certain pesticides. A suspension of two parts water to one part soil (2:1 ratio) was prepared and allowed to stand at room temperature for one (1) hour. The pH was then be determined using a manual pH meter or robotic system. Soil pH was measured in water, macro- and micro-nutrients were determined using modified Morgan extractant.

### 2.6. Fruit Quality and Bitter Pit Evaluation

Over three years (2016–2018) and each orchard, three weekly picks of commercial-grade fruit were made at harvest with the initial pick commencing at the time of the conventional commercial first harvest for each region. Forty-five apples were harvested at each pick. Fruit selection criteria for each pick mirrored the common commercial practice of spot-picking based on the visual intensity of the red blush. A 15-apple subsample was randomly selected and evaluated for external and internal fruit quality and maturity (FQM) parameters. Apple dimensions such as diameter (FD) and length (L) were measured using a digital caliper. Fruit shape or sphericity was then calculated as FD/L ratio. Fruit weight (FW) was determined by a digital scale. Red color coverage (% blush) was visually scored as a percentage in 10% increments. External ground color from both cheeks (CIELAB coordinates L, a, b) was determined using a Minolta Chroma Meter CR-200 portable tristimulus colorimeter (Minolta Corp, Osaka, Japan) in 2016, and an Agrosta Texture Analyzer (ver. 2016, Serqueux, FRA) in 2017 and 2018 measuring RGB then converted via algorithm to CIELAB L, a, b coordinates. Flesh firmness (FF), expressed in Newtons, was determined with a firmness tester (EPT, Lake City Technical Products, USA in 2016 and an Agrosta Texture Analyzer (ver. 2016, Serqueux, FRA) in 2017 and 2018, using an 11 mm diameter tip. Two readings were taken from opposite peeled sides of each fruit. Soluble solids content (SSC) and titratable acidity (TA) were determined using juice extracted with an automatic juicer (Maverick). SSC was determined using a digital hand-held refractometer (Atago Pal-1, Tokyo, Japan), with the results presented as °Brix. TA was determined by titrating 5 mL of juice with 0.1 sodium hydroxide (NaOH) to an end point of pH 8.2, and the results were expressed as g malic acid 100 mL^−1^ (Semi-Automatic Titrator HI94532U-O1, Hanna Instruments, Smithfield, RI, USA). The ripening index (RI) was determined as SSC/TA ratio. The starch pattern index (SPI) was determined for each fruit according to the Cornell 8-step scale published by Blanpied and Silsby [21].

The remaining thirty apples were stored in regular atmosphere at 2.2 °C for 120 days (without conditioning at 10 °C). Each of the 30 apples were labelled with a unique number and data specific to that fruit was collected whenever practical with non-destructive measurements made as described for the 15 apple FQM subsamples. BP incidence and % surface area in 10% increments was rated at harvest and at 60 and 120 days post-harvest. At 60 days, individual apple data was collected on BP severity by counting lesions. In addition, at 120 DAH (days after harvest), color and FF were evaluated at the individual apple level, SSC and TA were evaluated at the group level.

### 2.7. Statistical Analysis

The combinations of year and region and year and rootstock were built to evaluate their effect on all traits studied in this study. The t-Student and HSD Tukey tests were used to compare means at the 5% confidence level. Pearson correlation coefficients were determined to study correlations among traits. Partial Least Square Regression models (PLSR) were run to correlate weather, soil, horticultural and fruit quality traits as X-variables, and bitter pit incidence after 120 days of storage as response variable (Y-variable), to find the variables that had most weight for discriminating among regions and rootstocks for ‘Honeycrisp’. The non-linear iterative partial least squares (NIPALS) algorithm was used. The Leave-One-Out validation was used to select the number of factors. The function yields two metrics: the variable importance in the projection (VIP) and the regression coefficient. The VIP indicates the weighted sum of squares of the PLS loadings and represents the relative importance of the given predictor (X-variable) in the PLS model. VIP values above a fixed threshold value of 0.8 are considered significant [22]. The sign of the regression coefficient indicates the direction of the relationship between an X-variable and the Y-variable, while the magnitude of the regression coefficient reflects the VIP and is proportional to the contribution of all X-variables to bitter pit incidence at 120 days of storage. In addition, loadings were also calculated and plotted to give another way to view the relationships between the Xs the Ys and the PLSR factors. Whenever practical for data collected and recorded at the individual apple level, binomial bitter pit incidence data was analyzed directly for treatment differences using the Analysis of Means of Proportions (AMP) procedure. Continuous fruit weight and firmness data, when collected and recorded at the individual apple level, was analyzed directly for treatment differences using the Analysis of Means Methods (AMM) procedure. AMP and AMM results were presented in graphical form as well as in tables. All statistics analysis were performed using JMP software (Version 14, SAS Institute Inc., Cary, NC, USA). Bitter pit incidence data was arc-sin square root transformed and the non-normal distributions of average length terminal shoot and BP lesion density were rank transformed.

## 3. Results

To achieve at least three orchards per rootstock category (B.9, M.26 and M.9 clone) with satisfactory standard grower production practices and good fruit set, a total of 30 orchards were included in our final evaluation. ‘Honeycrisp’ orchards on MM.106, B.118, G.30 and EM.7 were excluded. Statistical analysis showed that year, region and their interaction, and year, rootstock and their interaction significantly affected some of the traits evaluated in this study.

### 3.1. The Effect of Region and Rootstock on Bitter Pit Incidence and Severity

**Figure 2:**
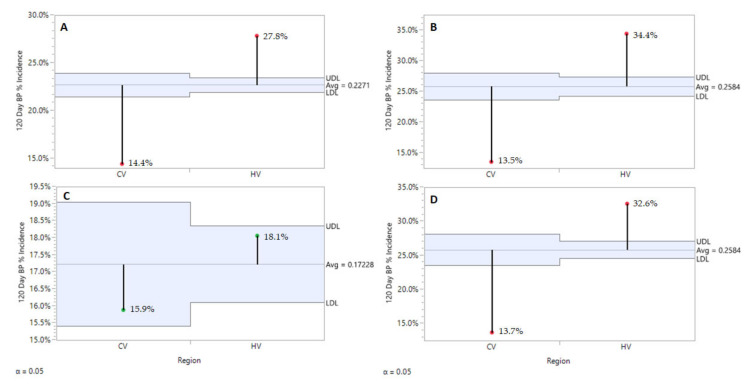
Region effect on ‘Honeycrisp’ bitter pit incidence after 120 days of refrigerated storage with all rootstocks and years combined (**A**), in 2016 with all rootstocks combined (**B**), in 2017 with all rootstocks combined (**C**), and in 2018 with all rootstocks combined. JMP Fit XY Platform, Analysis of Means of Proportions of the binomial dataset, alpha = 0.05.

**Figure 3:**
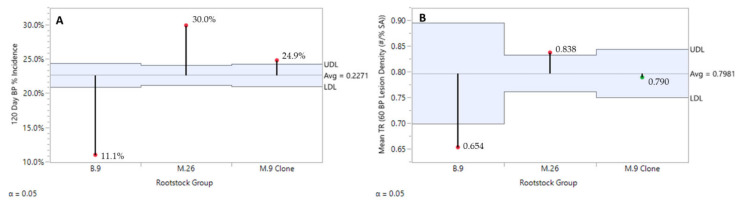
Rootstock effect on ‘Honeycrisp’ bitter pit incidence (**A**) and on ‘Honeycrisp’ bitter pit severity (**B**) after 120 days of refrigerated storage with all years and both regions combined (A). JMP Fit XY Platform, Analysis of Means of Proportions of the binomial dataset, alpha = 0.05.

**Figure 4:**
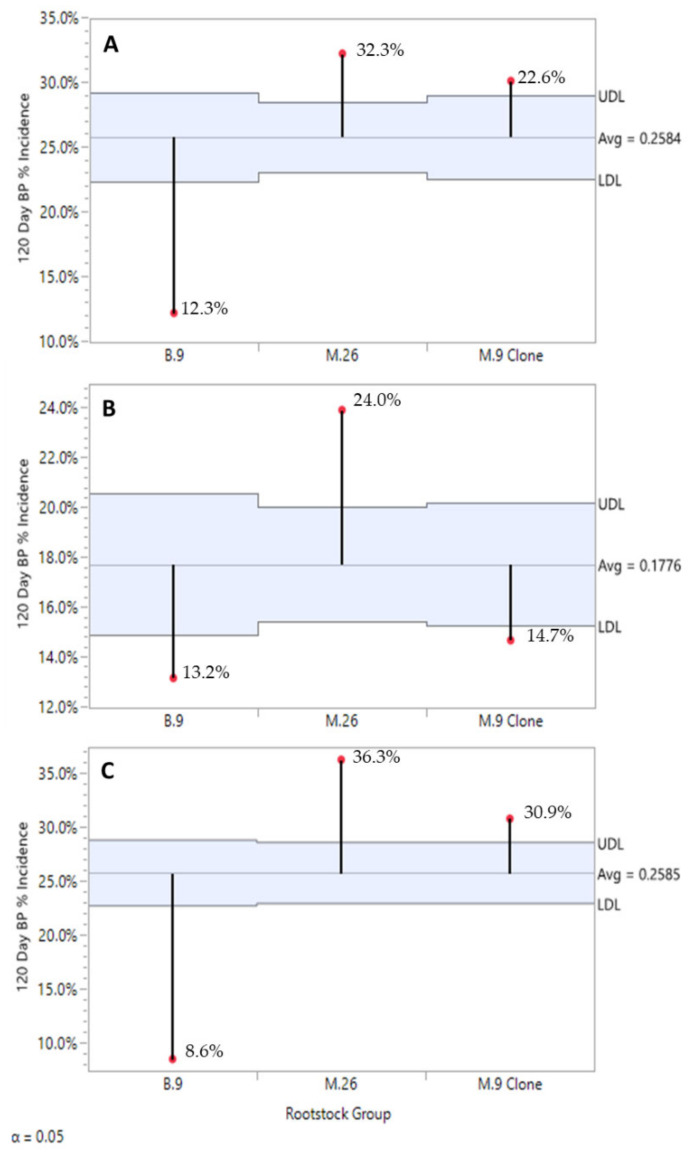
Rootstock effect on ‘Honeycrisp’ bitter pit incidence after 120 days of refrigerated storage in 2016 (**A**), in 2017 (**B**), and in 2018 (**C**) with both regions combined. JMP Fit XY Platform, Analysis of Means of Proportions of the binomial dataset, alpha = 0.05.

From a regional perspective, the Champlain Valley (CV) consistently produced fruit with significantly lower BP incidence in 2016 and 2018, and still numerically lower in 2017 while not statistically significant (Figure 2). Rootstock significantly affected BP incidence post-harvest (Figure 3A) and severity (Figure 3B) after 120 days of refrigerated storage for all years and regions combined. Individual ‘Honeycrisp’ fruits produced on the B.9 rootstock were much less likely to express bitter pit symptoms (11.1%) compared to M.9 (24.9%) and M.26 (30.0%) with the expression of symptoms on affected fruits being less severe as well. For both regions combined, each year B.9 fruit consistently expressed the least BP, M.26 fruit the highest, with M.9 found to be more variable in annual response, approaching B.9 levels of incidence in 2017 (Figure 4).

### 3.2. Weather and Soil Conditions on Each Region and Rootstock

Experimental units evaluated in this study over three consecutive years exhibited variability for all weather and soil traits evaluated (Table 2). In terms of year, region and their interaction, in 2016, ‘Honeycrisp’ orchards accumulated more degree days (DD) until harvest and had higher soil moisture compared to the same orchards in 2017 and 2018. Orchards from Hudson Valley (HV), in general, accumulated almost 24% and 11% more rain and DD that those from Champlain Valley (CV), but 11% less DD during the 60 day period post-bloom (Table 2). In addition, soil from HV orchards, in general, had more available water as well as lower pH, reduced LOI and organic matter content than those from CV (Table 2). 

Analyzing year, rootstock, and their interaction, in 2016 ‘Honeycrisp’ orchards accumulated more degree days (DD) until harvest and had higher soil moisture compared to the same orchards in 2017 and 2018 (Table 2). Among rootstocks, orchards from ‘Honeycrisp’ trees on M.9 clones (M.9-T337, NIC29, Pajam2) received more rain and degree days (DD) compared to those on M.26 and B.9 orchards, but less DD from 60 days post-bloom to harvest and soil pH (Table 2). In general, the variability found on all weather and soil traits was reflected in our finding no significant correlations with bitter pit (BP). All ‘Honeycrisp’ orchards from HV had no significant correlations between weather and soil traits and BP (Table 3). ‘Honeycrisp’ orchards from CV had a moderate negative correlation with soil sand and a moderate positive correlation between soil silt and BP. Regarding rootstock, the traits evaluated had no correlation with BP in B.9 orchards, but this was not the case in M.26 and M.9 orchards where some traits were found to be correlated (Table 3). In M.26 orchards, soil sand and soil silt had a moderate to low negative and moderate positive correlation with BP, respectively, whereas M.9 clone orchards had moderate to low negative correlation between soil sand and BP and moderate positively correlation between soil clay and BP. 

### 3.3. The Effect of Region and Rootstock on Selected Horticultural Parameters 

Year, region, and their interaction significantly affected some traits (Table 4). ‘Honeycrisp’ trees from HV were more vigorous, and had higher crop load (CL), leaf Ca, leaf Mg, leaf Mn, peel K, peel Mn and peel *p* values compared to those from CV, especially those in 2017 for leaf elements and those in 2016 for peel elements (Table 4). 

In terms of year, rootstock, and their interaction, the first two significantly affected some traits (Table 4). As expected, ‘Honeycrisp’ trees on M.26 had larger trunk diameters and lower absolute crop load (CL) expressed as number of fruits per cm^2^ trunk cross-sectional area (TCSA) compared to the M.9 clone and B.9 trees. M.26 trees produced numerically more terminal shoot growth (264.5 mm) than M.9 (225.8 mm) and B.9 (226.7 mm), but the differences were not statistically significant (Table 4). The effect of rootstock on leaf and peel mineral concentrations are also described in Table 4. Rootstock had a significant effect on the concentration of some minerals, mainly in leaf tissue. ‘Honeycrisp’ trees on M.26 had higher leaf K/Ca, Mg/Ca, B/Ca ratios, as well as K values alone than those on B.9 and M.9 clone rootstocks, but lower Ca, Mn, and P values. In terms of peel minerals, only the K/Ca ratio and B were significantly affected by rootstock, where the M.9 clones had the highest and the lowest values, respectively.

Multivariate analyses of all horticultural traits were carried out by region and rootstock for all years together (Table 5). For CV ‘Honeycrisp’ orchards, BP incidence was positively correlated with vigor, leaf Mg, peel K/Ca, Mg/Ca and B/Ca ratios, and negatively correlated with leaf Mn and peel Ca. Orchards from the HV had more horticultural parameters correlated with BP, including one with an opposite trend. BP incidence was positively correlated with vigor, ALTS, leaf and peel K/Ca, Mg/Ca, and B/Ca ratios, leaf K, peel B, peel K, peel Mg, and peel P, and negatively correlated with CL, leaf Ca, leaf Mg, leaf Zn and peel Ca (Table 5).

Different relationships were found when BP was tested against all horticultural traits for each rootstock (Table 5). For ‘Honeycrisp’ grafted on B.9, BP incidence after 120 days of storage was positively associated with vigor, and peel Mg/Ca, and negatively associated with CL and peel Ca. Different results were found for those fruits from M.26 and M.9 clonal rootstocks. For M.26, BP incidence was positively correlated with TCSA, leaf P, as well as all peel minerals evaluated and ratios, except for a negative correlation with Ca and no correlation with Zn (Table 5). Regarding M.9 clone orchards, BP incidence was positively correlated with leaf and peel K/Ca ratio, Mg/Ca ratio, and B/Ca ratio, and peel B, K, and P, but negatively correlated with leaf Ca and Zn, and peel Ca (Table 5). B/Ca ratio, peel Mg/Ca ratio and peel Ca correlated with BP for all three rootstocks, with the strongest correlations associated with the M.9 clone rootstocks (Table 5).

### 3.4. The Effect of Region and Rootstock on Select Fruit Quality Parameters 

For each year of the study, quality and maturity parameters of fruit harvested to a commercial standard in three weekly picks were evaluated at harvest and after 120 days at 2.2 °C without conditioning (Table 6). Regarding year, region, and year × region interaction, almost all traits evaluated were significantly affected (Table 6). In 2016, ‘Honeycrisp’ fruits were less elongated and firm, but sweeter and redder than those picked in 2017 and 2018, even after 120 days after storage (Table 6). Based on region, ‘Honeycrisp’ fruits from CV were larger but more spherical, firmer, sweeter, and redder than those from HV (Table 6). Rootstock affected significantly fewer fruit quality and maturity parameters than did region. With 10,895 apples from both regions over three years individually weighed, we found B.9 (235.1 g), M.9 clone (233.8 g) and M.26 (230.2 g) to be approximately the same size by weight, but those from B.9 and M.9 clone were less elongated (Table 6) Overall, fruits from ‘Honeycrisp’ trees on B.9 and M.9 clones had similar fruit maturity, except that SSC and blush values were lower in fruit from M.9 clones than B.9 (Table 6). 

Regarding year × rootstock interaction, overall ‘Honeycrisp’ on B.9 in 2016 had better commercial characteristics at harvest than the rest of combinations, especially for FD, SSC, and blush traits (Table 6). Multivariate analysis for BP against fruit quality traits at harvest and after storage for each rootstock category and for each region are presented in Table 7. BP after storage correlated positively with FD and FW for both M.26 and M.9 clone rootstocks. L/FD and blush ratio were positively and negatively correlated with BP incidence, respectively, for fruits on the M.26 rootstock, whereas SSC after storage was positively correlated with BP for B.9 fruits (Table 7). For orchards from the CV, BP was negatively correlated with blush, whereas it was positively correlated with FD, L/FD, and FW. From HV orchards, the fruit size variables FD, L/FD, FW and the fruit quality parameter TA (after storage) were correlated positively with BP incidence (Table 7).

### 3.5. The Effect of Pick Timing on Bitter Pit Incidence by Region and Rootstock 

**Figure 5:**
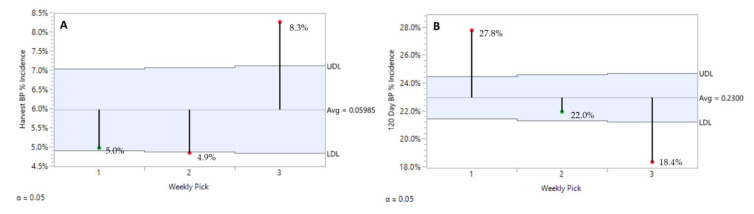
Pick timing effect on ‘Honeycrisp’ bitter pit incidence at harvest (**A**) and after 120 days of refrigerated storage (**B**) with all rootstocks and years combined. JMP Fit XY Platform, Analysis of Means of Proportions of the binomial dataset, alpha = 0.05.

For each experimental unit, ‘Honeycrisp’ fruits were picked three times on a weekly schedule, each year. At harvest, BP symptom expression was minimal with the first two picks at 5.0% and 4.9%, increasing to 8.3% for the third pick. Although these differences are minimal, they are statistically significant due to the large number of observations analyzed, but not likely to be detected by commercial operators in the field (Figure 5A). 

**Figure 6:**
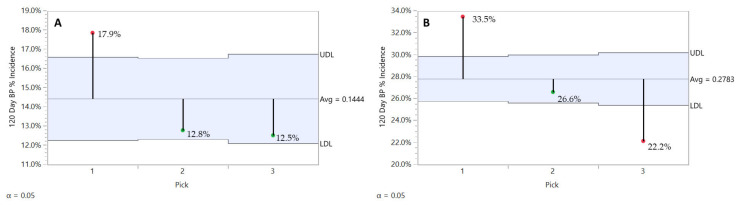
Pick timing effect on CV ‘Honeycrisp’ orchards (**A**) and HV ‘Honeycrisp’ orchards (**B**), bitter pit incidence after 120 days of refrigerated storage with all years combined. JMP Fit XY Platform, Analysis of Means of Proportions of the binomial dataset, alpha = 0.05.

In contrast, after 120 days of storage, ‘Honeycrisp’ fruits harvested at the first pick had the highest BP incidence (27.8%), followed by those from the second (22.0%) and the third (18.4%) (Figure 5B). Second and third pick showed significantly lower rates of BP symptom expression. There was a regional effect observed as well. For all rootstocks and years combined, BP in the CV decreased after pick 1, stabilizing with picks 2 and 3 (Figure 6A). In the HV, BP incidence decreased with each subsequent pick (Figure 6B). 

**Figure 7:**
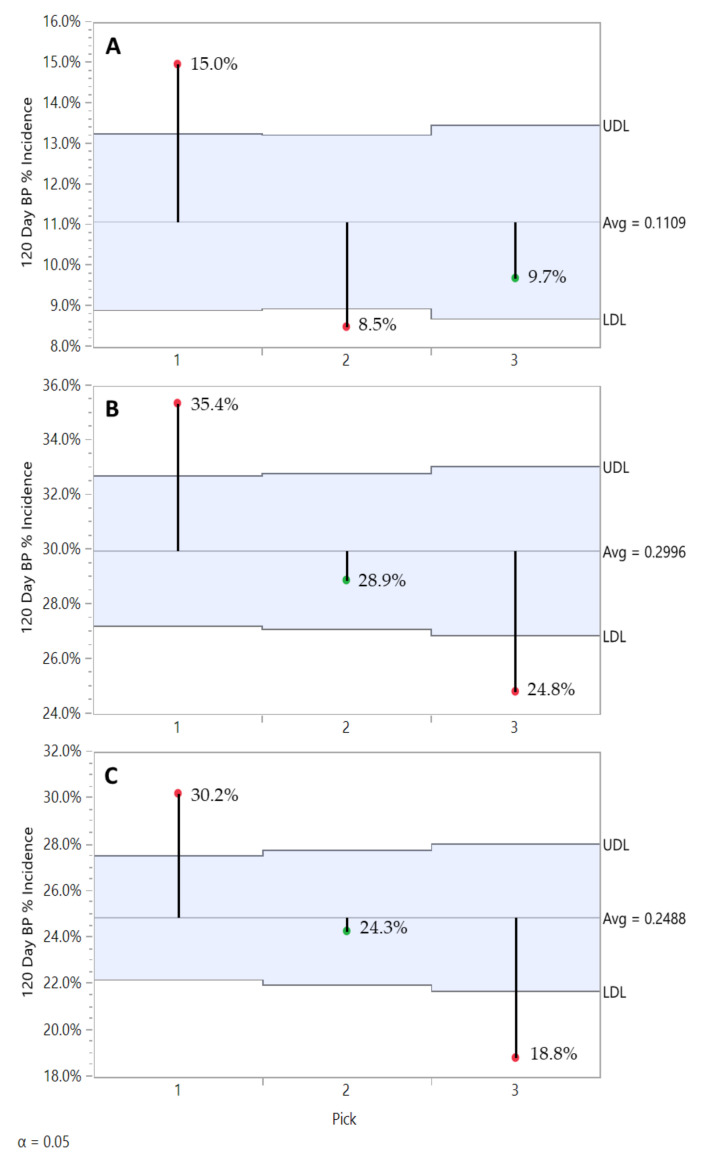
Pick timing effect on B.9 (**A**), M.26 (**B**), and M.9 Clone (**C**) ‘Honeycrisp’ orchards bitter pit incidence after 120 days of refrigerated storage with all years and regions combined. JMP Fit XY Platform, Analysis of Means of Proportions of the binomial dataset, alpha = 0.05.

We observed a rootstock effect on BP related to pick timing (Figure 7). First pick fruits from all three rootstocks had the highest BP incidence values, but in general B.9 fruit had lower values compared to the M.26 and M.9 clones, which had less than M.9 clone ‘Honeycrisp’ fruits. BP incidence in B.9 fruit for picks 2 and 3 was found to have stabilized at a level below that of pick 1 (Figure 7A). On the other hand, M.26 (Figure 7B) and M.9 clone (Figure 7C) fruit BP incidence followed a decreasing trend for picks 2 and 3. However, M.9 clone ‘Honeycrisp’ fruits from pick 3 in good conditions would offer better BP performance in storage. 

### 3.6. The Effect of Pick Timing on Fruit Quality Traits

**Figure 8:**
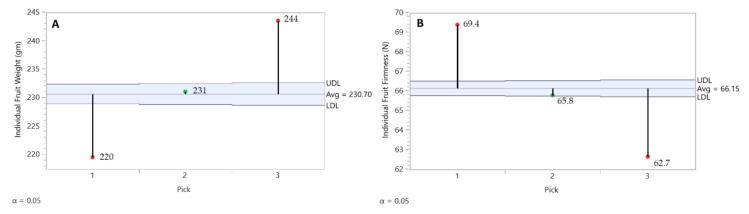
Picking time effect at harvest on ‘Honeycrisp’ fruit weight (**A**) and flesh firmness (**B**) with all rootstocks and years combined. As expected, fruit size increased with each subsequent pick, and fruit firmness decreased. JMP Fit XY Platform, Analysis of Means of Proportions of the binomial dataset, alpha = 0.05.

As was BP incidence, FW and FF were evaluated individually by fruit and followed through storage, which allowed us to study these two traits by pick. In general, ‘Honeycrisp’ fruits harvested at pick 1 were smaller but firmer than those harvested at pick 2, which at the same time were smaller and firmer than those harvest at pick 3 (Figure 8A,B). Therefore, fruits from pick 3 were 11% larger than those of pick 1, but BP incidence was consistently less. Across all picks (P.1, P.2, P.3), SSC (13.2, 13.2, 13.4), and % coverage of red blush (62.1, 65.2, 66.0) remained relatively stable, while % TA (0.606, 0.541, 0.498) decreased with later picks. The SSC/TA ratio increased only slightly between picks 1 and 2, but substantially increased in pick 3 fruit (22.7, 23.6, 28.0). At harvest, the average starch pattern index (SPI) ranged from 6.5 for pick 1, 7.1 for pick 2, and 7.2 for pick 3 on the Cornell 8-step scale. Pick 3 fruit firmness and SPI (Cornell 1-8 scale) were similar to the fruit harvested by Watkins and Nock [23] for their study of ‘Honeycrisp’ controlled atmosphere storage quality, while SSC (13.4% vs. 11.4%) and TA (0.498 vs. 0.303) were considerably higher than the fruit in that study, suggesting that our pick 3 fruit were suitable for commercial acceptance in the marketplace.

### 3.7. The Effect of Fruit Size on Bitter Pit Incidence by Rootstock 

**Figure 9:**
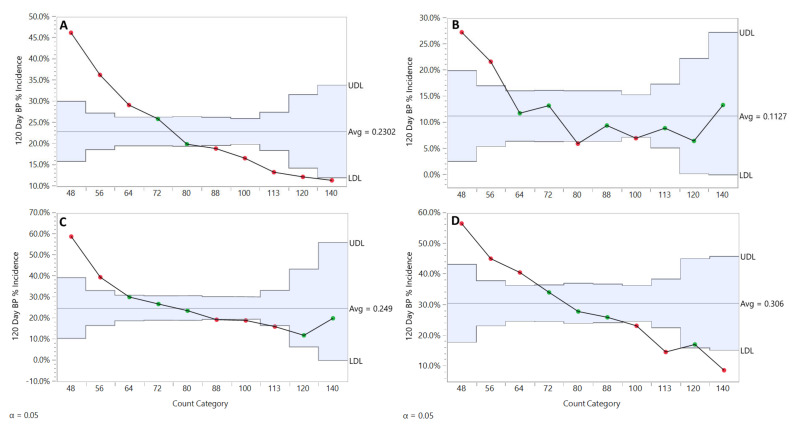
‘Honeycrisp’ bitter pit incidence after 120 days storage by count size category, all rootstocks, regions, and years (**A**), and by B.9 (**B**), M.26 (**C**) and M.9 clone (**D**) all regions and all years. JMP Fit XY Platform, Analysis of Means of Proportions of the binomial dataset, alpha = 0.05.

“Count size” is a construct used by the commercial fruit industry in the United States to categorize tray packs of individual fruits by size. The term “count” refers to the number of apples that will fill a standard 18.2 kg cardboard tray-pack box used in the wholesale marketing chain. Each apple in our database was assigned to a “count size category” based on weight according to a commercially acceptable [24] scheme as implemented by a commercial apple packer in the HV. For all rootstocks in all regions for all years we found a very clear relationship between apple size and bitter pit incidence that varied by size category. BP incidence was found to increase in a near linear manner through the commonly marketed count categories 140 through 64, with a strong increase observed in the very large 48 and 56 count sizes (Figure 9A). These sizes are difficult to market, and often sold as low value cullage. The largest fruit had 4.5-fold the susceptibility for BP than the smallest fruit evaluated in this study.

When analyzed by rootstock, we found differences in BP susceptibility as it relates to fruit size. B.9 fruit are less susceptible to BP in general, and while the large 48 and 56 count sizes showed the characteristic uptick, BP incidence across the remaining commercially viable sizes was found to be relatively flat with some random fluctuation (Figure 9B). Fruits from the M.9 clones (Figure 9C), and M.26 groups (Figure 9D) consistently showed a decline in BP incidence with decreasing size, the trend being much more pronounced for M.26. The data suggests that very large fruit are highly susceptible to BP when produced on all rootstocks. In the range of commercially marketable sizes, B.9 appears to have a stabilizing effect on the relationship between fruit size and BP susceptibility that was not observed in the M.9 clones and M.26 fruit.

### 3.8. Partial Least Square Analysis for Bitter Pit in Relation to All Traits Evaluated by Region and Rootstock

To determine the variables that most influenced BP, two PLSR models were run. An initial PLSR model was developed by analyzing all years together for each region and rootstock category using all variables above mentioned as the X variables and BP incidence at 120 DAH as the Y variable to eliminate some of the noise. Following the preliminary PLSR regression the important Xs variables (VIP > 0.8) were identified and included in the final PLSR prediction model for each region and rootstock category. CL variable was not included in the PLS analysis, 2018 data was not taken as it is shown in Table 3.

**Figure 10:**
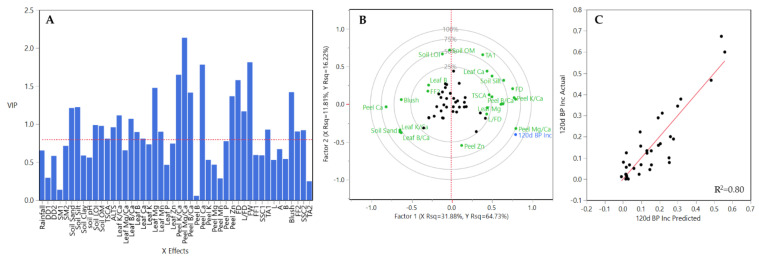
Results obtained from the partial least square (PLS) analysis between BP incidence at 120 DAH and the rest of variables evaluated all three years and all rootstocks together for CV. (**A**) Important X variables in the PLS model, (**B**) correlation loading plot, and (**C**) observed values versus PLSR-predicted values for BP. Abbreviations: ALTS: average length terminal shoot; DD1: degree days accumulated from 1st of January to harvest date; DD2: degree days accumulated from 60 days post-bloom; FD: fruit diameter; FF1: flesh firmness at harvest; FF2: flesh firmness after storage; FW: fruit weight; L: length; SSC1: soluble solids content at harvest; SSC2: soluble solids content after storage; SM1: average soil moisture 12 weeks prior to harvest; SM2: average soil moisture from 1st of January to harvest date; TA1: titratable acidity at harvest; TA2: titratable acidity after storage; TCSA: trunk cross-sectional area.

Between regions (CH and HV), they showed a different response to the PLSR model prediction. For CV, 22 out of 46 Xs variables were excluded (VIP <0.08) for the final PLSR model. The remaining variables, soil sand, soil silt, soil LOI, soil OM, TCSA, ALTS, leaf K/Ca, leaf B/Ca, leaf B, leaf Ca, leaf Mg, leaf Mn, peel K/Ca, peel Mg/Ca, peel B/Ca, peel Ca, peel P, peel Zn, FD, L/FD, FW, TA1, blush, FF2, and SSC2 were included in the final PLSR model (Figure 10A). From these variables peel K/Ca, peel Mg/Ca, peel Ca and FW had the highest coefficient values (Figure 10A). The correlation loading plot (factor 1 and factor 2) explained 43.69% of the variation (Figure 9B). Among Xs variables, leaf K/Ca, leaf B/Ca, peel Mg/Ca, peel Ca, among others contributed greatly to explained variation. A strong relationship was observed between BP incidence-predicted values and the observed ones (Figure 10C). 

**Figure 11:**
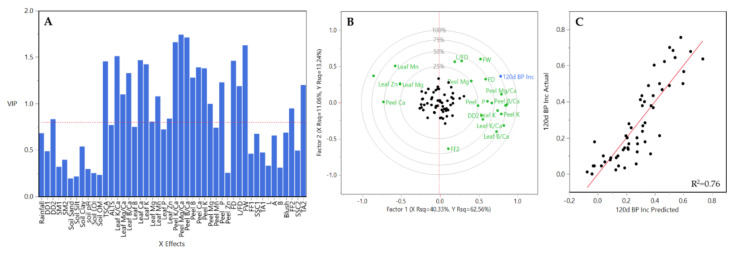
Results obtained from the partial least square (PLS) analysis between BP incidence at 120 DAH and the rest of variables evaluated all three years and all rootstocks together for HV. (**A**) Important X variables in the PLS model, (**B**) correlation loading plot, and (**C**) observed values versus PLSR-predicted values for BP. Abbreviations: ATS: average length terminal shoot; DD1: degree days accumulated from 1st of January to harvest date; DD2: degree days accumulated from 60 days post-bloom; FD: fruit diameter; FF1: flesh firmness at harvest; FF2: flesh firmness after storage; FW: fruit weight; L: length; SSC1: soluble solids content at harvest; SSC2: soluble solids content after storage; SM1: average soil moisture 12 weeks prior to harvest; SM2: average soil moisture from 1st of January to harvest date; TA1: titratable acidity at harvest; TA2: titratable acidity after storage; TCSA: trunk cross-sectional area.

In the case of HV region, 23 out of 46 Xs variables were included in final PLSR model (Figure 11A). These variables were DD2, TCSA, leaf K/Ca, leaf Mg/Ca, leaf B/Ca, leaf Ca, leaf K, leaf Mg, leaf Mn, leaf Zn, all peel elements except for Mn and Zn, FD, L/FD, FW, FF2, and TA2 were included in the final PLSR model (Figure 11A). From these variables peel K/Ca, peel Mg/Ca and peel B/Ca had the highest coefficient values (Figure 9A). The correlation loading plot (factor 1 and factor 2) explained 51.39% of the variation (Figure 11B). Among Xs variables, leaf Zn, peel Mg/Ca, peel B/Ca, and others contributed greatly to explained variation. A strong relationship was observed between BP incidence-predicted values and the observed ones (Figure 11C).

**Figure 12:**
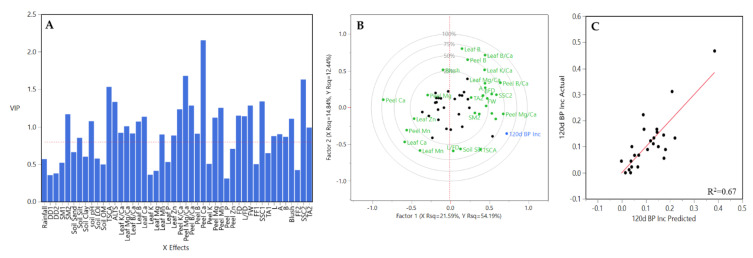
Results obtained from the partial least square (PLS) analysis between BP incidence at 120 DAH and the rest of variables evaluated all three years together for B.9 rootstock in HV and CV. (**A**) Important X variables in the PLS model, (**B**) correlation loading plot, and (**C**) observed values versus PLSR-predicted values for BP. Abbreviations: ALTS: average length terminal shoot; DD1: degree days accumulated from 1st of January to harvest date; DD2: degree days accumulated from 60 days post-bloom; FD: fruit diameter; FF1: flesh firmness at harvest; FF2: flesh firmness after storage; FW: fruit weight; L: length; SSC1: soluble solids content at harvest; SSC2: soluble solids content after storage; SM1: average soil moisture 12 weeks prior to harvest; SM2: average soil moisture from 1st of January to harvest date; TA1: titratable acidity at harvest; TA2: titratable acidity after storage; TCSA: trunk cross-sectional area.

In the case of the B.9 rootstock, preliminary PLSR regression removed 17 out of 46 Xs variables with minor contribution (VIP < 0.08) to the prediction model (Figure 12A). The remaining variables were included in the final PLSR model. These variables were SM2, soil silt, soil pH, TCSA, ALTS, leaf K/Ca, leaf Mg/Ca, leaf B/Ca, leaf B, leaf Ca, leaf Mn, leaf Zn, peel K/Ca, peel Mg/Ca, peel B/Ca, peel B, peel Ca, peel Mg, peel Mn, FD, L/FD, FW, SSC1, L, A, B, blush, SSC2 and TA2. The amount of peel Ca, peel Mg/Ca, SSC2 and TA were among the most powerful variation X variables in the preliminary PLSR model. The above X dataset (17 Xs variables) was reduced to two principal factors (Figure 12B). The first explained the 21.59% of the variation while the second explained the 14.84%. Thus, the accumulated variation explained by two principal factors was 37.43%. Among X variables peel Mg/Ca, peel B/Ca, leaf B/Ca, peel Ca, leaf Ca, leaf B, and peel B, were the greatest contributors to explained variation. In terms of Y data, two factors explained 66.63% of the variation, with the first contributing with 54.19% and the second with 12.44%. Predicted BP incidence values derived from PLSR model were plotted over the observed values showing a good relationship (Figure 12C).

**Figure 13:**
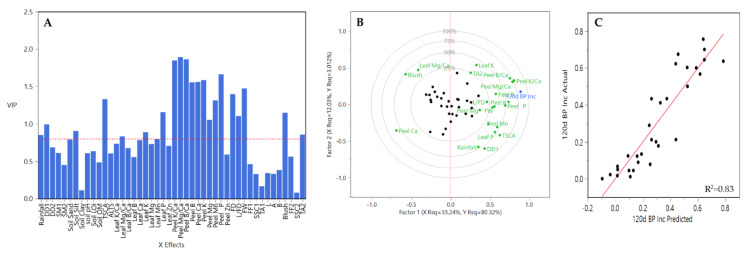
Results obtained from the partial least square (PLS) analysis between BP incidence at 120 DAH and the rest of variables evaluated all three years together for M.26 rootstock in HV and CV. (**A**) Important X variables in the PLS model, (**B**) correlation loading plot, and (**C**) observed values versus PLSR-predicted values for BP. Abbreviations: ALTS: average length terminal shoot; DD1: degree days accumulated from 1st of January to harvest date; DD2: degree days accumulated from 60 days post-bloom; FD: fruit diameter; FF1: flesh firmness at harvest; FF2: flesh firmness after storage; FW: fruit weight; L: length; SSC1: soluble solids content at harvest; SSC2: soluble solids content after storage; SM1: average soil moisture 12 weeks prior to harvest; SM2: average soil moisture from 1st of January to harvest date; TA1: titratable acidity at harvest; TA2: titratable acidity after storage; TCSA: trunk cross-sectional area.

A different trend was observed for M.26 rootstock. Fewer and different Xs variables contributed to the prediction model (21 out of 46 Xs variables) (Figure 13A). These variables were rainfall, DD1, soil silt, TCSA, leaf Mg/Ca, leaf K, leaf Mn, leaf P, peel K/Ca, peel Mg/Ca, peel B/Ca, peel Ca, peel K, peel Mg, peel Mn, peel P, FD, L/FD, FW, blush, and TA2. Among them, peel K/Ca, peel Mg/Ca, and peel B/Ca had the greater coefficient values across Y prediction model, followed by peel B, peel Ca, and peel K (Figure 13A). Factor 1 explained 33.24% of the variation and factor 2 the 12.03%, being peel K/Ca, peel Mg/Ca, peel B/Ca, peel Ca, peel P, TCSA, and DD1, the greatest contributors to explained variation (Figure 13B). A strong relationship was observed between BP incidence-predicted values and the observed ones (Figure 13C).

**Figure 14:**
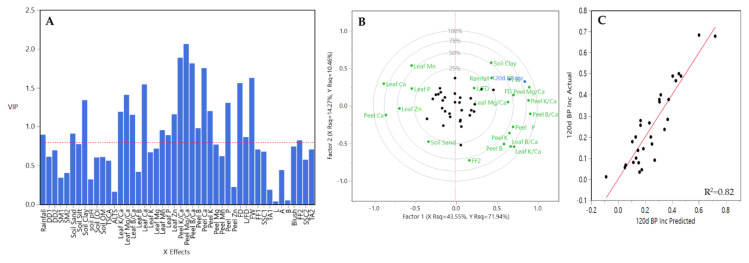
Results obtained from the partial least square (PLS) analysis between BP incidence at 120 DAH and the rest of variables evaluated all three years together for M.9 clone rootstocks in HV and CV. (**A**) Important X variables in the PLS model, (**B**) correlation loading plot, and (**C**) observed values versus PLSR-predicted values for BP. Abbreviations: ALTS: average length terminal shoot; DD1: degree days accumulated from 1st of January to harvest date; DD2: degree days accumulated from 60 days post-bloom; FD: fruit diameter; FF1: flesh firmness at harvest; FF2: flesh firmness after storage; FW: fruit weight; L: length; SSC1: soluble solids content at harvest; SSC2: soluble solids content after storage; SM1: average soil moisture 12 weeks prior to harvest; SM2: average soil moisture from 1st of January to harvest date; TA1: titratable acidity at harvest; TA2: titratable acidity after storage; TCSA: trunk cross-sectional area.

Regarding M.9 clone rootstocks, 21 out of 46 Xs variables were included in final PLSR model (Figure 14A). They were rainfall, soil sand, soil clay, leaf K/Ca, leaf Mg/Ca, leaf B/Ca, leaf Ca, leaf Mn, leaf P, leaf Zn, peel K/Ca, peel Mg/Ca, peel B/Ca, peel B, peel Ca, peel K, peel P, FD, L/FD, FW, FF2. The highest coefficient values were for peel K/Ca, peel Mg/Ca, peel Ca and FW, (Figure 14A). The two principal factors for X data accounted 57.82% of the variation and for Y data accounted 82.40%, with the first contributing with 43.55% and 71.94% and the second with 14.26% and 10.46%, respectively. Among X variables peel B/Ca, peel K/Ca, peel Mg/Ca and peel Ca were the greatest contributors to explained variation (Figure 14B). A strong relationship was observed between BP incidence-predicted values and the observed ones (Figure 14C).

Finally, comparing the results obtained for each PLSR prediction model, very few Xs variables with VIP values above 0.8 were in common among them. The variables in common were peel K/Ca, peel Mg/Ca, and peel B/Ca ratios, peel Ca, FD, L/FD, and FW. 

## 4. Discussion 

In the course of this work, we evaluated a high number of parameters as possible indicators of BP incidence, including weather and soil traits, horticultural and fruit quality characteristics, through the perspective of region and rootstock choice, by conducting a detailed survey of 34 ‘Honeycrisp’ blocks distributed across two growing regions in Eastern NY, which at the end totaled 30 blocks. Our goal was to describe as much of the biological and abiotic world that our 6-tree experimental units were expected to thrive in while producing marketable fruit in commercial settings. The authors can say with confidence that the commercial producers who donated their orchards to this study were among the most skilled in New York State, with well-managed ‘Honeycrisp’ plantings. 

Regional and local environmental and soil conditions must be taken in consideration when planting a new orchard and may be significant contributors to BP predisposition. To the best of our knowledge, this is the first study evaluating the region effect on the occurrence of BP. After three years and comparing the two regions, we found that, in general, ‘Honeycrisp’ orchards from the HV region presented high BP incidence. This region received more rain and experienced higher temperatures over the study period, which may explain partially the difference in BP. 

In addition to region, rootstock choice is one of the most critical elements of any apple orchard to provide sufficient growth control, enhanced precocity, higher yield, improved adaptability to environmental conditions, and better fruit quality [25]. In addition to effects on these traits, apple rootstocks have a diverse influence on the nutritional status of the tree canopy, are implicated in the physiology of BP and, therefore, can affect the occurrence of BP [26,27,28], as it is demonstrated in our results. However, the BP response to tissue mineral status is variable depending on the rootstock and the region where it is planted. As a result, the occurrence of BP can be more or less intense or absent even as local tree tissue mineral measurements suggest otherwise.

We evaluated three of the most popular rootstocks used in high-density apple orchards in New York State: B.9, M.26 and M.9 clones [25]. Among them, fruits from ‘Honeycrisp’ grafted on M.26 were slightly more susceptible to BP than those from M.9 clones and much more susceptible than B.9. In agreement with Lordan et al. [28], B.9 rootstocks had a much lower incidence of BP compared to M.26 and M.9 clones, even in the very dry year of 2016. In general, B.9 BP incidence values did not differ significantly among years by region, even when both regions were evaluated together. Kim and Ko [29] reported that BP is more intensive on moderate, vigorous rootstocks compared to less vigorous rootstocks, which is consistent with our results, as M.26 is the most vigorous rootstock in terms of TCSA evaluated in this study. Terminal shoot extension was a poor indicator of vigor and BP incidence as ALTS was very similar between the three rootstocks while BP differed significantly.

In terms of horticultural parameters, region and rootstock had a significant effect on some of these traits, results that were somewhat expected. Other authors have also reported that region and rootstock can affect similar horticultural traits under Hudson Valley and Champlain Valley climatic conditions for ‘Gala’, ‘Fuji’ and ‘Honeycrisp’ [7,25,28]. In this study, the most vigorous rootstock, M.26, had higher leaf K/Ca, Mg/Ca and B/Ca ratios, leaf K, and peel B, but lower leaf Ca, Mn, and P values as compared to B.9 and M.9 clones. 

Between regions, ‘Honeycrisp’ orchards, despite showing significant differences, some of these traits were not correlated to BP incidence after a period of refrigerated storage. ‘Honeycrisp’ fruits from CV orchards tended to have less BP incidence after storage (less than 10%) compared to those from HV. This lower BP value may explain the lower number of correlations with the horticultural traits, as well as the higher BP incidence values of M.26 orchards from HV could explain the higher number of significant correlations with horticultural traits compared to those from CV region.

This is the first study reporting rootstock effect on correlations between BP and horticultural traits. Little correlation was found between BP incidence after storage on ‘Honeycrisp’ fruits from B.9 in terms of horticultural traits, TCSA, peel Mg/Ca and peel Ca, whereas more significant correlations were found in fruit from the M.26 and M.9 clones, mainly the peel minerals. The lower BP incidence values from B.9 fruits could explain the lack of correlations compared to M.26 and M.9 clone rootstocks. These two rootstocks had some correlations in common, such as peel K/Ca, peel Mg/Ca, peel B/Ca, peel B, peel Ca, peel K and peel P, but M.9 clone rootstocks had higher values. 

Recent studies have shown that BP, a Ca^2+^-related deficiency disorder, is not necessarily related to low Ca^2+^ concentration in fruit tissue in a “global” sense. In fact, chemical and X-ray analysis have shown that apple fruit tissue with visual Ca^2+^ deficiency symptoms had higher Ca^2+^ concentration than healthy fruit tissue [30]. Most Ca^2+^ in fruit tissue, between 60 and 75%, is bound to the cell wall. More Ca^2+^ binding to the cell wall is consistent with the finding that BP-damaged tissues have more Ca^2+^ than the surrounding healthy tissues [31,32]. In agreement with this statement and previous studies [3,33], we found a high and negative correlation between peel Ca^2+^ concentration and BP incidence after storage for all three rootstock categories and two regions.

Fruit quality traits were also affected by region and rootstock, in agreement with previous rootstocks studies performed in ‘Gala’, ‘Fuji’, ‘Honeycrisp’ and ‘Red Delicious’ under Hudson Valley and Champlain Valley climatic conditions [7,25,28,34]. Both regions (CV and HV) had similar correlations between fruit dimensions and BP incidence after storage, despite showing significantly differences on these traits. However, blush only correlated with BP on those ‘Honeycrisp’ from CV. BP incidence after storage had few and inconsistent correlations with fruit dimensions and fruit quality traits when rootstocks were compared. ‘Honeycrisp’ fruits from M.26 rootstock, which had in general smaller FD because they were more elongated but similar FW to B.9 and M.9 clones, presented a moderate positive correlation with BP incidence after storage on these three parameters, and a medium negative correlation with blush. In contrast, B.9 did not present any correlation on the same traits, while M.9 clones did in FD and FW, perhaps this finding is associated with lower levels of BP and less variability in the B.9 orchards. A similar trend was observed regionally for B.9.

‘Honeycrisp’ fruits were harvested at optimum commercial harvest quality at each of the three weekly picking times. Minor fruit quality and maturity differences between picks at harvest were found but considered to be commercially acceptable for storage and marketing purposes. BP incidence at the time of harvest was relatively low and varied only slightly by pick with the pick 3 (last pick) apples expressing slightly more BP. It would be unlikely for a commercial producer to observe the slight uptick in BP in the field. In contrast, BP incidence after storage showed a significant decreasing trend in each of the later picks in the HV, while in the lower BP environment of the CV, picks 2 and 3 were found to be similar, and lower than pick 1. 

‘Honeycrisp’ fruits picked earlier were firmer, smaller, with more red blush and presented higher BP in storage. Therefore, in agreement with Prange et al. [35], BP is more severe in early-picked than in later-picked apples. However, there may be an optimum stage of fruit maturity (or harvest date) for ‘Honeycrisp’ when fruit are of sufficient size and color to meet market requirements while minimizing the risk of manifesting BP, especially if the fruit are >250 g in size. Our study did not attempt to specifically evaluate that possibility. We closely adhered to commonly accepted commercial quality standards. In any case there may not be much room available to adjust harvest dates and maintain a balance of quality factors acceptable to the marketplace. 

Increasing fruit size has been associated with increased BP incidence [36]. The relationship was further defined by Reid and Kalcsits [37] in a water relations study where fruit size was categorized into four classes based on diameter, with BP incidence effectively doubling between the 80–90 mm and over 90 mm categories. Our study takes this approach a step further, with the use of ten commercial weight categories in the range of 48 count (largest) down to 140 count (smallest) based on common marketing practice. For all storage fruit in this study the frequency distribution of across the ten categories approximated the bell shape of a normal distribution with the top of the “bell” flattened (data not shown), with 92% of the fruit falling into count categories 56 to 113. For all three rootstocks, fruit in the categories 48 and 56 were the most susceptible to BP. While our categories were based on weight ranges, our fruit diameter data shows that 48 count apples averaged 94.1 mm and 56 count apples averaged 89.3 mm, both categories roughly equivalent to the largest size category described in the Reid and Kalcsits [37] study which also experienced an elevated incidence of BP. The relationships start to change by rootstock as we move into the more commonly marketed size categories. Fruit produced on B.9 had a relatively neutral relationship of BP to size in the range from 64 to 140 as the BP incidence curve flattened and oscillated around a mean of 11.2% incidence. Fruit produced on M.9 demonstrated a decline in BP incidence with decreasing size, with incidence falling from 29.2% (64 count) to 13.3% (113 count). Fruit produced on M.26 demonstrated the most severe relationship falling from 40.6% to 14.6% over the same count size range. There are orchard management implications associated with these findings. As much as the industry recognizes that larger fruit have more bitter pit, as a practical matter the first priority of a properly managed crop load reduction program is to produce fruit in marketable sizes, and then facilitate adequate return bloom to avoid biennial bearing. Minimizing the production of 48 and 56 count apples will have a positive effect on orchard financial returns for all rootstocks represented in this study. Beyond that, a shift in frequency distribution to smaller fruit is not likely to help in a B.9 orchard and will only slightly reduce the average BP incidence in M.9 clone and M.26 orchards.

While BP incidence has been related to individual mineral element concentrations and ratios of mineral pairs in many apple studies, one should not underestimate the complex environment that the roots (soil type, soil pH, water availability, soil moisture, etc.), and the scion (rainfall, light intensity, crop load, heat unit accumulation) operate in, in conjunction with the final fruit traits influence by producer management practices during the course of the dormant and growing seasons. For this reason, we pooled together all the traits evaluated in this study, except for CL, which was not evaluated in 2018, to identify the PLS prediction model on BP for each region and each rootstock based on the NIPALS algorithm.

Based on the results, the PLS prediction model for each region (CV and HV) and each rootstock (B.9, M.26 and M.9 clone) showed a different threshold of variables correlated to BP, described above for each PLS prediction model. However, comparing all PLS analysis, only seven VIP variables were in common, peel K/Ca, peel Mg/Ca, and peel B/Ca ratios, peel Ca, FD, L/FD, and FW, showing the great variability found in this study. It is also interesting to point out that none of the environmental variables and soil variables evaluated in this study were VIP variables in common among rootstocks or between regions. The 34 orchards evaluated in this study over three years represent a wide range of these variables, therefore, these results could help to emphasize their influence on BP incidence when taking in consideration each rootstock and each region as a single unit to evaluate.

The results of this work have the potential for a dramatic impact on commercial management and mitigation of BP in ‘Honeycrisp’ production. In order to facilitate real-time management changes, producers and marketers need practical tools and proven horticultural practices that mitigate bitter pit incidence and reduce storage decision risk. Bitter pit prediction models are currently in various stages of development, validation, and commercial implementation [12,16,38] with all three taking different approaches to meet the same goal of reliable pre-harvest prediction of ‘Honeycrisp’ fruit BP performance in storage. Recommended approaches should be on those that are simple to implement at a low cost to the producer. However, the large number of variables suggests that simple and commercially achievable models consisting of 1–3 variables will always be lacking in absolute accuracy. Fortunately for practical implementation within the apple industry, accuracy thresholds for commercial implementation are more tolerant of error than those considered acceptable in academic settings. The goal is to provide effective storage management guidance which ultimately protects the producer from making the unprofitable decision to store fruit from an orchard that turns out to suffer substantial losses to BP months later.

## 5. Conclusions

Our findings on the role of region and rootstock choice in BP mitigation have strong commercial consequences. We suggest that the BP performance of a rootstock should be a major consideration when choosing a rootstock for a new ‘Honeycrisp’ orchard in New York State and likely elsewhere as well. Unfortunately, data beyond anecdotal observations is difficult to find, and considering the variability found in this study, likely to be highly unreliable. We suggest that rootstocks newly introduced to the commercial market should be tested for BP performance during the developmental phase and before being recommended for widespread use with ‘Honeycrisp”, beyond the scope of modest producer test plantings. 

In a more basic sense, these results could also suggest that in addition to the variables considered in this study, and commonly studied in others, there are other, less studied factors or triggers (genetic, histological, hormonal, abiotic stress situations, etc.) that can influence the physical expression of BP symptoms. With that said, identifying and understanding these factors may help to uncover the mechanism within the tree associated with the fruit, maintaining an adequate supply of calcium cations in the vicinity of groups of cells, making sure that they are available at the appropriate time, and what factors or combinations of factors influence the effectiveness of this calcium delivery mechanism, if possible. 

## Figures and Tables

**Table 1:** Site descriptions.

Region	Orchard	Farm	Elevation (m)	Rootstock	Rootstock Category	Soil Texture	Soil Water Capacity	Soil Aggregate Stability
Hudson Valley (HV)	1	Porpiglia	136	M.9-337	M.9 Clone	Silt loam	0.298	23.1
	2	Porpiglia	127	M.9-337	M.9 Clone	Sandy loam	0.186	19.2
	3	MG Hurd	176	M.9-337	M.9 Clone	Loam	0.225	13.5
	4	WG Minard	133	M.9-337	M.9 Clone	Silt loam	0.243	47.9
	5	WG Minard	127	M.9-337	M.9 Clone	Silt loam	0.236	48.5
	6	Crist Bros	151	M.26	M.26	Loam	0.178	5.2
	7	Crist Bros	149	B.9	B.9	Loam	0.278	11.0
	8	Crist Bros	158	M.26	M.26	Loam	0.214	29.1
	9	Mead	65	B.9	B.9	Loam	0.180	69.0
	10	Yonder	82	B.9	B.9	Silt loam	0.197	43.4
	11	Bartolotta	75	M.26	M.26	Silt loam	0.195	39.8
	12	Bartolotta	91	M.9-337	M.9 Clone	Loam	0.181	25.8
	13	Fix Bros	77	B.9	B.9	Silt loam	0.205	32.2
	14	Fix Bros	58	Pajam2	M.9 Clone	Sandy loam	0.156	23.5
	15	Yonder	91	M.26	M.26	Sandy loam	0.105	17.9
	16	Yonder	88	NIC29	M.9 Clone	Sandy loam	0.127	12.6
	17	Yonder	93	M.9-337	M.9 Clone	Loam	0.187	15.5
	18	Yonder	98	M.26	M.26	Silt loam	0.194	22.6
	19	Saulpaugh	78	M.26	M.26	Sandy loam	0.127	30.7
	20	Saulpaugh	72	MM.106	Other	Sandy loam	0.120	45.9
Champlain Valley (CV)	21	Chazy	49	M.9-337	M.9 Clone	Silt loam	0.168	46.2
	22	Chazy	38	B.118	Other	Silt loam	0.194	44.9
	23	Chazy	33	B.9	B.9	Loamy sand	0.085	52.9
	24	Chazy	60	B.9	B.9	Silt loam	0.209	28.8
	25	Forrence	121	M.26	M.26	Loam	0.177	10.7
	26	Forrence	119	M.26	M.26	Sandy loam	0.113	19.9
	27	Forrence	48	M.26	M.26	Loam	0.132	46.8
	28	Forrence	56	M.26	M.26	Sandy loam	0.126	48.5
	29	Northern	134	B.9	B.9	Loam	0.143	28.3
	30	Northern	128	M.26	M.26	Loam	0.156	37.2
	31	Northern	185	G.30	Other	Loam	0.138	18.3
	32	Hart	127	EM.7	Other	Loam	0.174	36.3
	33	Hart	127	B.9	B.9	Loam	0.134	17.7
	34	Hart	127	B.9	B.9	Loam	0.154	31.2

**Table 2:** Weather and soil conditions for all three years of study (2016, 2017 and 2018) and all three rootstocks (B.9, M.26 and M.9 Clone) from both regions (CV and HV).

Trait	Rainfall ^a^	DD1	DD2	SM1	SM2	Soil Sand	Soil Silt	Soil Clay	Soil pH	Soil LOI	Soil OM
Year (Y)											
2016	841.8	2878.0 a	853.6 b	47.0 a	33.7 a	45.4	40.5	14.0	6.6	4.3	2.8 a
2017	902.3	2748.7 b	808.2 c	22.2 b	22.7 b	45.1	40.8	14.1	6.7	4.3	2.8 a
2018	891.7	2687.5 c	890.4 a	32.4 b	29.2 ab	45.1	40.8	14.1	6.5	4.0	2.3 b
*Significance*	*0.4026*	*<0.0001*	*<0.0001*	*0.0002*	*0.0277*	*0.9948*	*0.9938*	*0.9995*	*0.6325*	*0.5672*	*0.0391*
Region (RE)											
CV	783.9 b	2632.2 b	894.7 a	46.5 a	38.1 a	49.1 a	37.1 b	13.7	6.8 a	4.6 a	2.8 a
HV	973.3 a	2910.7 a	897.2 b	21.2 b	18.5 b	41.3 b	44.3 a	14.4	6.4 b	3.8 b	2.4 b
*Significance*	*<0.0001*	*<0.0001*	*<0.0001*	*<0.0001*	*<0.0001*	*0.0031*	*0.0016*	*0.2882*	*0.0223*	*0.0060*	*0.0245*
Y × RE											
2016 CV	701.9 c	2691.3 c	907.2 a	68.8 a	46.8	49.1	37.2	13.7	6.8	4.8	3.1
2016 HV	981.7 a	3064.7 a	800.0 c	25.2 bc	20.6	41.7	43.9	14.4	6.4	3.8	2.5
2017 CV	878.1 abc	2628.9 c	864.2 b	23.1 bc	27.6	49.1	37.1	13.7	6.9	4.6	3.0
2017 HV	926.4 ab	2868.5 b	753.4 d	21.4 c	17.8	41.1	44.4	14.4	6.5	3.9	2.5
2018 CV	771.6 bc	2576.3 c	912.6 a	47.7 ab	41.1	49.1	37.2	13.7	6.7	4.3	2.3
2018 HV	1011.8 a	2798.8 b	868.3 b	16.9 c	17.2	41.1	44.4	14.4	6.4	3.7	2.2
*Significance*	*0.0388*	*0.0069*	*<0.0001*	*0.0017*	*0.0969*	*0.9950*	*0.9930*	*0.9998*	*0.9366*	*0.7778*	*0.4508*
Year (Y)											
2016	879.5	2917.4 a	842.2 b	41.3 a	30.4	44.4	41.4	14.2	6.6	4.2	2.7
2017	908.3	2786.0 b	792.7 c	22.0 b	21.3	43.7	42.0	14.2	6.6	4.2	2.7
2018	919.7	2715.1 b	884.8 a	28.7 ab	26.4	43.9	41.9	14.2	6.5	3.9	2.3
*Significance*	*0.7127*	*<0.0001*	*<0.0001*	*0.0173*	*0.1581*	*0.9722*	*0.9667*	*0.9966*	*0.5978*	*0.5784*	*0.0514*
Rootstock (R)											
B.9	838.6 a	2744.0 b	860.1 a	34.4	30.1	43.7	41.5	14.8	6.5 ab	4.5 a	2.8 a
M.26	879.1 ab	2782.9 b	847.6 a	34.2	27.4	47.1	39.1	13.8	6.8 a	3.7 b	2.3 b
M.9 Clone	989.8 a	2891.7 a	812.1 b	23.5	20.5	41.3	44.7	14.0	6.3 b	4.1 ab	2.6 ab
*Significance*	*0.0069*	*0.0021*	*0.0002*	*0.1741*	*0.1152*	*0.1699*	*0.1116*	*0.4205*	*0.0044*	*0.0350*	*0.0486*
Y × R											
2016 B.9	854.5	2820.2	869.4	42.4	32.6	44.9	40.3	14.7	6.5	4.4	2.8
2016 M.26	841.8	2886.4	851.3	55.7	37.9	47.1	39.1	13.8	6.9	3.9	2.5
2016 M.9 Clone	942.3	3045.7	806.0	25.8	20.7	41.3	44.7	14.0	6.2	4.4	2.8
2017 B.9	890.4	2776.9	814.1	23.5	24.6	42.7	42.3	14.9	6.8	5.1	3.3
2017 M.26	901.4	2764.8	802.4	22.8	22.4	47.1	39.1	13.8	6.8	3.6	2.3
2017 M.9 Clone	933.2	2816.4	761.7	19.9	16.9	41.3	44.7	14.0	6.3	4.0	2.6
2018 B.9	771.0	2634.9	896.8	37.1	33.2	43.4	41.8	14.7	6.3	4.1	2.3
2018 M.26	894.0	2697.4	889.0	24.1	21.9	47.1	39.1	13.8	6.8	3.7	2.1
2018 M.9 Clone	1093.9	2812.9	868.6	24.9	23.9	41.3	44.7	14.0	6.4	4.0	2.4
*Significance*	*0.2147*	*0.4916*	*0.7624*	*0.2593*	*0.4418*	*0.9987*	*0.9981*	*1.000*	*0.5327*	*0.4993*	*0.4636*

Means followed by the same letter in each column are not significantly different at *p* ≤ 0.05 according to the t-Student and HSD Tukey tests. Abbreviations: DD1: degree days accumulated from 1st of January to harvest date; DD2: degree days accumulated from bloom to 60 days post-bloom; LOI: mass loss on ignition; OM: organic matter; SM1: average soil moisture 12 weeks prior to harvest in centibars; SM2: average soil moisture from 1st of January to harvest date in centibars. ^a^ Annual rainfall accumulated from 1st of January until harvest date.

**Table 3:** Multivariate analysis for bitter pit (%) in ‘Honeycrisp’ apples stored at 2.2 °C for 120 days against all weather and soil traits for each rootstock category and region with all three years combined (2016–2018).

Trait	Region		Rootstock		
	CV	HV	B.9	M.26	M.9 Clone
Rainfall	ns	ns	ns	ns	ns
DD1	ns	ns	ns	ns	ns
DD2	ns	ns	ns	ns	ns
SM1	ns	ns	ns	ns	ns
SM2	ns	ns	ns	ns	ns
Soil Sand	−0.40 *	ns	ns	−0.34 *	−0.37 *
Soil Silt	0.41 *	ns	ns	−0.40 *	ns
Soil Clay	ns	ns	ns	ns	0.53 **
Soil pH	ns	ns	ns	ns	ns
Soil LOI	ns	ns	ns	ns	ns
Soil OM	ns	ns	ns	ns	ns

* *p* < 0.05; ** *p* < 0.01; ns, not significant. Abbreviations: DD1: degree days accumulated from 1st of January to harvest date; DD2: degree days accumulated from bloom to 60 days post-bloom; LOI: mass loss on ignition; OM: organic matter; SM1: average soil moisture 12 weeks prior to harvest; SM2: average soil moisture from 1st of January to harvest date. Annual rainfall accumulated from 1st of January until harvest date.

**Table 4:** Horticultural measurements of ‘Honeycrisp’ for year, region, rootstock, and their interactions.

Trait	Final TCSA	CL	ALTS	Leaf										Peel									
				K/Ca	Mg/Ca	B/Ca	B	Ca	K	Mg	Mn	P	Zn	K/Ca	Mg/Ca	B/Ca	B	Ca	K	Mg	Mn	P	Zn
Year (Y)																							
2016	40.5	2.9	210.3	1.2 ab	0.26 a	0.003	41.7 a	12553.9 b	14582.6 a	3029.5 a	59.7 b	1754.3	22.1	22.2 a	2.1	0.07	27.6	408.3 a	9103.4 a	809.6 b	6.9	813.4 ab	3.2 b
2017	44.3	2.9	242.9	0.9 b	0.20 b	0.003	34.9 a	15568.7 a	11428.3 b	3007.2 ab	104.6 a	1815.8	23.7	16.2 b	1.9	0.06	24.5	439.0 a	6655.5 c	794.6 b	7.4	729.6 b	5.2 ab
2018	43.1	-	260.0	1.3 a	0.23 ab	0.004	34.0 b	11836.6 b	13172.9 a	2525.1 b	61.1 b	1633.3	25.6	21.1 a	2.3	0.07	27.9	446.9 a	7956.8 b	915.6 a	7.7	839.3 a	6.9 a
*Significance*	0.9326	0.9772	0.1211	0.0078	0.0192	0.0678	<0.0001	0.0009	<0.0001	0.0236	<0.0001	0.2190	0.6186	0.0100	0.0959	0.3158	0.1278	0.3505	<0.0001	0.0001	0.1859	0.0144	0.0002
Region (RE)																							
CV	33.4 b	2.3 b	252.7 a	1.3 a	0.23	0.004	35.6	12197.8 b	14020.9 a	2583.9 b	41.9 b	1765.8	21.1	15.6 b	1.9 b	0.06	26.6	455.3 a	6622.7 b	834.7	6.6 b	740.5 b	5.8
HV	51.8 a	3.6 a	227.9 a	1.0 b	0.22	0.003	38.1	14441.7 a	12101.7 b	3124.1 a	108.3 a	1703.1	26.4	24.1 a	2.2 a	0.07	26.8	407.5 b	9187.8 a	845.1	8.1 a	847.7 a	4.4
*Significance*	0.0325	0.0166	0.2281	0.0024	0.9183	0.1135	0.0501	0.0075	0.0004	0.0018	<0.0001	0.4696	0.0742	<0.0001	0.0175	0.0923	0.9204	0.0411	<0.0001	0.6679	<0.0001	0.0009	0.0585
Y × RE																							
2016 CV	30.9	2.8 ab	226.4 ab	1.1 a	0.24 a	0.002 b	33.5 bc	13789.9 b	14367.7 ab	3107.0 ab	43.7 c	1839.3	21.3 ab	17.9 b	1.9 b	0.06 ab	23.8 bc	434.2 abc	7398.6 c	792.5	6.3	699.5 c	2.7 b
2016 HV	50.1	3.0 ab	194.1 b	1.4 a	0.26 a	0.005 a	49.8 a	11317.8 b	14797.6 a	2952.1 ab	75.6 bc	1669.3	22.9 ab	26.4 a	2.2 ab	0.08 a	31.5 a	382.4 bc	10808.2 a	826.7	7.6	927.4 a	3.7 b
2017 CV	35.6	1.7 b	301.0 a	1.3 a	0.22 a	0.004 ab	35.7 bc	11445.4 b	13259.8 ab	2414.5 b	46.3 bc	1770.9	15.2 b	16.7 b	2.1 ab	0.07 ab	28.2 ab	402.9 abc	6206.9 c	814.7	6.8	704.5 bc	6.3 ab
2017 HV	52.7	4.2 a	212.4 ab	0.5 b	0.18 a	0.002 b	34.1 bc	19691.9 a b	9596.9 c	3600.0 a	162.9 a	1860.6	32.1 a	15.8 b	1.7 b	0.05 b	20.9 c	475.1 ab	7104.2 c	774.5	8.1	754.6 bc	4.1 b
2018 CV	33.4	-	230.5 ab	1.6 a	0.23 a	0.005 a	37.6 b	11357.9 b	14435.2 ab	2230.1 b	35.7 c	1687.2	26.8 ab	12.3 b	1.7 b	0.05 b	27.9 ab	528.7 a	6262.6 c	896.9	6.7	817.7 abc	8.5 a
2018 HV	52.7	-	277.0 ab	1.1 a	0.24 a	0.003 b	30.5 c	12315.2 b	11910.7 b	2820.2 b	86.4 b	1579.4	24.4 ab	30.1 a	2.8 a	0.09 a	27.8 ab	365.1 c	9651.2 b	934.2	8.7	861.0 ab	5.4 ab
*Significance*	0.9912	0.0324	0.0295	0.0009	0.1067	<0.0001	<0.0001	<0.0001	0.0062	0.0070	0.0002	0.4511	0.0245	<0.0001	<0.0001	0.0001	0.0004	0.0004	<0.0001	0.3467	0.5010	0.0268	0.0493
Year (Y)																							
2016	40.8	2.9	202.8 a	1.2 a	0.26 a	0.004 a	43.4 a	12346.8 b	14467.9 a	3002.3 ab	62.5 b	1742.2	22.2	22.9 a	2.1 ab	0.07 a	28.4 a	402.9	9430.9 a	809.4 b	7.1	838.7 ab	3.3 b
2017	44.7	3.4	242.7 a	0.8 b	0.19 b	0.002 b	34.8 b	16733.9 a	10899.8 c	3140.1 a	119.8 a	1840.1	25.9	16.1 b	1.8 b	0.05 b	23.3 b	451.8	6788.3 b	787.7 b	7.6	741.9 b	4.7 ab
2018	44.1	-	257.9 a	1.2 a	0.23 ab	0.003 ab	33.0 b	12141.8 b	12811.8 b	2607.5 b	68.2 b	1631.1	25.8	23.3 a	2.4 a	0.07 a	27.8 a	425.3	8373.2 a	918.7 a	7.9	844.5 a	6.5 a
*Significance*	0.8993	0.4063	0.0757	0.0011	0.0013	0.0206	<0.0001	<0.0001	<0.0001	0.0388	<0.0001	0.1186	0.4939	0.0033	0.0158	0.0393	0.0093	0.2982	<0.0001	<0.0001	0.1421	0.0243	0.0018
Rootstock (R)																							
B.9	25.5 b	3.2 ab	213.8	0.9 b	0.19 b	0.003 b	36.8	14881.8 a	12128.5 b	2690.6	71.9 b	1903.6 a	27.2	17.3 b	1.9	0.06	26.5 ab	452.0	7559.5	822.2	7.3	819.9	4.9
M.26	70.6 a	2.0 b	263.9	1.5 a	0.25 a	0.004 a	38.9	11845.6 b	13915.1 a	2857.6	73.4 b	1642.8 b	21.8	20.8 ab	2.1	0.07	29.9 a	439.4	8449.1	876.5	7.5	814.7	4.8
M.9 Clone	33.6 b	4.3 a	225.8	1.0 b	0.23 a	0.003 b	35.5	14495.2 a	12135.9 b	3201.7	105.3 a	1666.9 ab	25.0	24.2 a	2.3	0.07	23.1 b	388.6	8583.8	817.2	7.9	790.5	4.8
*Significance*	<0.0001	0.0008	0.1041	<0.0001	0.0007	0.0050	0.1931	0.0101	0.0080	0.0603	0.0178	0.0250	0.3205	0.0194	0.1039	0.3356	0.0006	0.0893	0.0741	0.0600	0.4494	0.7491	0.9936
Y × R																							
2016 B.9	24.3	3.0	172.9	1.0	0.23	0.003 ab	40.4	13375.2	12806.9	2911.6	49.0	1856.9	21.1	18.8	1.9	0.07	27.6	422.4	8447.9	763.9	6.6	812.1	2.9
2016 M.26	67.1	2.3	247.4	1.4	0.26	0.004 ab	43.3	11756.1	15976.4	3069.1	64.2	1727.9	22.6	23.3	2.1	0.07	30.7	416.3	9899.9	843.5	7.1	852.4	3.5
2016 M.9 Clone	30.9	3.6	188.0	1.3	0.26	0.004 ab	46.5	11909.2	14620.5	3026.0	74.4	1641.6	22.8	26.5	2.3	0.08	27.0	370.1	9944.9	820.8	7.5	851.4	3.4
2017 B.9	27.1	3.4	251.6	0.7	0.17	0.002 b	35.7	16553.9	10826.4	2629.3	99.9	2001.9	24.8	15.6	1.8	0.06	24.1	470.9	6751.4	797.2	7.5	800.7	4.9
2017 M.26	72.3	1.8	266.9	1.1	0.23	0.003 ab	36.7	14393.5	12278.7	3082.5	101.2	1712.4	23.8	16.5	1.9	0.07	27.8	444.3	6902.9	824.1	7.5	727.2	4.7
2017 M.9 Clone	34.9	5.0	209.7	0.6	0.19	0.002 b	31.9	19254.2	9594.4	3708.5	158.3	1806.1	29.3	16.1	1.7	0.04	17.9	440.1	6710.4	741.9	7.8	697.8	4.5
2018 B.9	25.1	-	216.8	0.9	0.18	0.002 b	34.3	14716.1	12752.4	2530.8	66.7	1852.0	25.8	17.3	2.0	0.06	27.7	462.7	7479.1	905.5	7.6	846.7	6.9
2018 M.26	72.3	-	277.4	1.8	0.27	0.005 a	36.6	9387.3	13490.8	2421.1	54.7	1488.0	18.8	22.8	2.4	0.08	31.3	457.5	8544.3	961.7	8.0	864.6	6.3
2018 M.9 Clone	34.9	-	279.7	1.0	0.24	0.002 b	28.1	12322.1	12192.9	2870.5	83.3	1553.0	22.9	29.9	2.8	0.08	24.3	355.5	9096.2	888.8	8.3	822.2	6.5
*Significance*	0.9998	0.2719	0.4834	0.1807	0.4302	0.0466	0.0539	0.1737	0.3430	0.3729	0.5173	0.8711	0.1748	0.3437	0.3125	0.4161	0.6402	0.7104	0.5950	0.6296	0.9903	0.7021	0.9842

Means followed by the same letter in each column are not significantly different at *p* ≤ 0.05 according to the t-Student and HSD Tukey tests. ALTS: average length terminal shoot; CL: crop load; TCSA: trunk cross-sectional area.

**Table 5:** Multivariate analysis for bitter pit (%) in ‘Honeycrisp’ apples stored at 2.2 °C for 120 days against all horticultural traits for each rootstock and region with all three years combined (2016–2018).

Trait	Region		Rootstock		
	CV	HV	B.9	M.26	M.9 Clone
Final TCSA	0.54 **	0.47 **	0.45 *	0.52 **	ns
CL	ns	−0.48 **	−0.61*	ns	ns
ALTS	ns	0.27*	ns	ns	ns
Leaf					
K/Ca	ns	0.61 ***	ns	ns	0.47 **
Mg/Ca	ns	0.41 **	ns	ns	0.59 **
B/Ca	ns	0.49 **	ns	ns	0.47 **
B	ns	ns	ns	ns	ns
Ca	ns	−0.56 ***	ns	ns	−0.64 ***
K	ns	0.58 ***	ns	ns	ns
Mg	0.45 **	−0.31*	ns	ns	ns
Mn	−0.38 *	ns	ns	ns	ns
P	ns	ns	ns	0.44 *	ns
Zn	ns	−0.31 *	ns	ns	−0.47 **
Peel					
K/Ca	0.67 ***	0.64 ***	ns	0.72 ***	0.79 ***
Mg/Ca	0.82 ***	0.66 ***	0.50 *	0.71 ***	0.85 ***
B/Ca	0.59 **	0.65 ***	ns	0.68 ***	0.76 ***
B	ns	0.50 ***	ns	0.57 **	0.37 *
Ca	−0.65 ***	−0.52 ***	−0.62 **	−0.57 **	−0.74 ***
K	ns	0.53 ***	ns	0.66 ***	0.50 **
Mg	ns	0.40 **	ns	0.39 *	ns
Mn	ns	ns	ns	0.52 **	ns
P	ns	0.50 ***	ns	0.66 ***	0.55 **
Zn	ns	ns	ns	ns	ns

* *p* < 0.05; ** *p* < 0.01; *** *p* < 0.001; ns, not significant. Abbreviations: ALTS: average length terminal shoot; CL: crop load; TCSA: trunk cross-sectional area.

**Table 6:** Fruit quality of ‘Honeycrisp’ from each year, region, rootstock category and their interactions at harvest and postharvest time.

Trait	At Harvest										After 120 Days of Storage		
	FD	L/FD	FW	FF	SSC	TA	L	A	B	Blush	FF	SSC	TA
Year (Y)													
2016	82.5 a	0.82 b	233.8	66.5 b	13.9 a	0.58 a	42.6 b	36.1 b	19.6 c	69.9 a	65.1 b	13.9 a	0.36 b
2017	80.3 b	0.85 a	235.4	67.6 a	13.1 b	0.59 a	24.2 c	42.8 a	29.3 b	68.5 a	67.2 ab	12.9 b	0.42 a
2018	79.7 b	0.85 a	233.7	65.7 c	13.2 b	0.44 b	52.1 a	13.3 c	53.2 a	65.2 b	67.7 a	11.7 c	0.39 ab
*Significance*	*<0.0001*	*<0.0001*	*0.7291*	*<0.0001*	*<0.0001*	*<0.0001*	*<0.0001*	*<0.0001*	*<0.0001*	*<0.0001*	*0.0472*	*<0.0001*	*0.0212*
Region (RE)													
CV	80.4 b	0.83 b	240.3 a	67.8 a	13.9 a	0.57 a	37.8 b	30.9	32.7 b	74.1 a	66.2	13.2 a	0.39
HV	81.3 a	0.85 a	228.3 b	65.3 b	12.9 b	0.50 b	41.4 a	30.5	35.3 a	61.8 b	67.1	12.6 b	0.38
*Significance*	*0.0002*	*0.0361*	*<0.0001*	*<0.0001*	*<0.0001*	*0.0004*	*<0.0001*	*0.5224*	*<0.0001*	*<0.0001*	*0.3039*	*0.0073*	*0.5997*
Y × RE													
2016 CV	83.7 a	0.81 d	242.3 ab	65.1 c	14.2 a	0.59	40.7 d	37.2 b	18.7 d	76.3 a	61.4 b	13.6 a	0.29 c
2016 HV	81.4 bc	0.83 cd	225.3 c	67.9 b	13.7 c	0.57	44.4 c	24.9 b	20.5 d	63.7 c	68.7 a	14.3 a	0.43 ab
2017 CV	78.6 d	0.86 ab	253.0 a	71.2 a	13.7 c	0.63	23.2 f	41.9 a	28.5 c	73.7 ab	68.4 a	13.6 a	0.48 a
2017 HV	82.1 b	0.85 abc	217.8 c	63.9 c	12.4 d	0.55	25.2 e	43.8 a	29.9 c	63.4 c	65.9 ab	12.3 b	0.36 bc
2018 CV	79.0 d	0.84 bcd	225.6 c	67.3 b	13.9 b	0.49	49.6 b	13.7 c	50.9 b	72.2 b	68.6 a	12.3 b	0.41 ab
2018 HV	80.4 c	0.87 a	241.9 b	64.2 c	12.5 d	0.38	54.6 a	12.9 c	55.3 a	58.3 d	66.7 a	11.2 c	0.36 bc
*Significance*	*<0.0001*	*0.0266*	*<0.0001*	*<0.0001*	*<0.0001*	*0.1160*	*0.0086*	*0.0253*	*0.0023*	*0.0082*	*<0.0001*	*0.0007*	*<0.0001*
Year (Y)													
2016	82.4 a	0.82 b	232.5	66.8 a	13.9 a	0.58 a	42.9 b	35.9 a	19.8 c	69.1 a	65.9	14.0 a	0.38
2017	80.9 b	0.85 a	230.7	66.5 a	12.9 c	0.58 a	24.6 c	43.1 a	29.5 b	66.9 b	66.9	12.7 b	0.40
2018	79.9 c	0.86 a	235.9	65.3 b	13.0 b	0.42 b	52.7 a	13.2 c	53.8 a	63.4 c	67.5	11.6 c	0.38
*Significance*	<0.0001	<0.0001	0.0646	<0.0001	<0.0001	<0.0001	<0.0001	<0.0001	*<0.0001*	*<0.0001*	*0.4062*	*<0.0001*	*0.5840*
Rootstock (R)													
B.9	81.1 b	0.83 b	235.1	66.7 a	13.5 a	0.53	39.7 b	30.9	34.2	68.8 a	67.4	12.9	0.37
M.26	80.2 c	0.85 a	230.2	65.6 b	13.2 b	0.52	39.5 b	30.3	33.9	67.4 a	65.8	12.7	0.39
M.9 Clone	81.9 a	0.83 b	233.8	66.4 a	13.1 b	0.53	40.9 a	31.0	34.9	63.2 b	67.1	12.8	0.39
*Significance*	*<0.0001*	*0.0148*	*0.0949*	*0.0009*	*<0.0001*	*0.8702*	*0.0050*	*0.5493*	*0.0666*	*<0.0001*	*0.3752*	*0.8156*	*0.4230*
Y × R													
2016 B.9	82.7 a	0.81	233.2 bc	66.0 bc	13.9 a	0.58	41.8	36.6	19.2	73.1 a	64.9	13.8	0.34
2016 M.26	82.2 ab	0.82	231.5 bc	65.3 cd	13.9 a	0.55	42.4	36.0	19.6	68.7 bc	64.3	13.9	0.37
2016 M.9 Clone	82.4 ab	0.82	232.8 bc	69.1 a	13.7 a	0.61	44.4	35.0	20.7	65.4 cd	68.5	14.3	0.42
2017 B.9	80.9 bcd	0.86	239.2 ab	67.9 ab	13.2 bc	0.58	24.7	43.3	29.7	66.6 bcd	68.3	13.1	0.39
2017 M.26	79.8 cd	0.87	231.5 bc	67.3 ab	12.9 d	0.59	24.0	42.5	29.1	69.2 b	66.8	12..9	0.43
2017 M.9 Clone	82.0 ab	0.83	221.4 c	64.4 d	12.6 e	0.58	24.9	43.6	29.7	65.0 d	65.5	12.4	0.39
2018 B.9	79.8 cd	0.84	232.7 bc	66.3 bc	13.4 b	0.43	52.5	12.9	53.8	66.8 bcd	68.9	11.7	0.37
2018 M.26	78.7 d	0.87	227.8 bc	64.3 d	12.6 e	0.42	52.2	13.3	53.0	64.3 d	66.5	11.3	0.38
2018 M.9 Clone	81.2 bcd	0.86	247.3 a	65.5 cd	13.0 cd	0.41	53.5	14.4	54.4	59.3 e	67.2	11.8	0.39
*Significance*	*0.0065*	*0.2175*	*<0.0001*	*<0.0001*	*<0.0001*	*0.7862*	*0.5151*	*0.4346*	*0.682*	*<0.0001*	*0.2083*	*0.3845*	*0.5743*

Means followed by the same letter in each column are not significantly different at *p* ≤ 0.05 according to the t-Student and HSD Tukey tests. Abbreviations: FD: fruit diameter; FF: flesh firmness; FW: fruit weight; L: length; SSC: soluble solids content; TA: titratable acidity.

**Table 7:** Bitter pit multivariate analysis in ‘Honeycrisp’ apples stored at 2.2 °C for 120 days against all fruit dimensions and fruit quality traits for each rootstock category over three years of study (2016–2018).

Trait	At Harvest										After 120 Days Storage		
	FD	L/FD	FW	F	SSC	TA	L	A	B	Blush	FF	SSC	TA
Rootstock													
B.9	ns	ns	ns	ns	ns	ns	ns	ns	ns	ns	ns	0.46 *	ns
M.26	0.42 *	0.45 **	0.47 **	ns	ns	ns	ns	ns	ns	−0.50 **	ns	ns	ns
M.9 Clone	0.65 ***	ns	0.67 ***	ns	ns	ns	ns	ns	ns	ns	ns	ns	ns
Region													
CV	0.54 **	0.37 *	0.60 **	ns	ns	ns	ns	ns	ns	−0.55 **	ns	ns	ns
HV	0.52 ***	0.37 *	0.57 ***	ns	ns	ns	ns	ns	ns	ns	ns	ns	0.49 **

* *p* < 0.05; ** *p* < 0.01; *** *p* < 0.001; ns, not significant. Abbreviations: FD: fruit diameter; FF: flesh firmness; FW: fruit weight; L: length; SSC: soluble solids content; TA: titratable acidity.

## Data Availability

Data is contained within the article.
